# Exosomal microRNAs shuttling between tumor cells and macrophages: cellular interactions and novel therapeutic strategies

**DOI:** 10.1186/s12935-022-02594-y

**Published:** 2022-05-16

**Authors:** Wen-Xiu Xu, Dan-Dan Wang, Zhi-Qiang Zhao, He-Da Zhang, Su-Jin Yang, Qian Zhang, Lei Li, Jian Zhang

**Affiliations:** 1 Department of General Surgery, The First Affiliated Hospital With Nanjing Medical University, 300 Guanzhou Road, Nanjing, 210029 China; 2 The Affiliated Huai’an Hospital of Xuzhou Medical University and The Second People’s Hospital of Huai’an, No. 62, Huaihai Road (S.), Huaian, 223002 China

**Keywords:** Extracellular vesicles, Tumor associated macrophages, Cancers, Tumorigenesis, Tumor microenvironment

## Abstract

Extracellular vesicles secreted by tumor microenvironment (TME) cells are vital players in tumor progression through transferring nucleic acids and proteins. Macrophages are the main immune cells in TME and tumor associated macrophages (TAM) express M2 phenotype, which induce tumor proliferation, angiogenesis, invasion, metastasis and immune elimination, resulting in the subsequent evolution of malignancies. There are a high number of studies confirmed that tumor cells and TAM interact with each other through extracellular vesicles in various cancers, like pancreatic ductal adenocarcinoma, gastric cancer, breast cancer, ovarian cancer, colon cancer, glioblastoma, hepatocellular cancer, and lung cancer. Herein, this review summarizes the current knowledge on mechanisms of communications between tumor cells and TAM via extracellular vesicles, mainly about microRNAs, and targeting these events might represent a novel approach in the clinical implications of this knowledge into successful anti-cancer strategies.

## Introduction

Extracellular vesicles are discoid, 30–150 nm sized and originate from endosomes [1]. They serve as a vehicle for genetic cargo and are able to deliver lipids, microRNAs (miRNAs), long non-coding RNAs (lncRNAs), mRNAs, DNAs, proteins and many other biomolecules [2–4]. Thus, extracellular vesicles act as intercellular messengers and significant mediators between cancer cells and tumor microenvironment, especially cancer cells and macrophages [5–7].

Macrophages can be polarized into classic M1 (antitumorigenic activity) and alternative M2 macrophages (protumorigenic activity) in response to various stimuli and cytokines [8]. M2-type macrophages produce high levels of anti-inflammatory factors like IL-10, IL-13 and IL-4 to facilitate tumor growth. Tumor associated macrophages (TAM) resemble M2-type and are the most abundant immune-related stromal cells within tumor microenvironment in various cancers, such as pancreatic ductal adenocarcinoma, gastric cancer, breast cancer, ovarian cancer, colon cancer, glioblastoma, hepatocellular cancer, and lung cancer [9–16]. Accumulating studies show that TAM promote cancer cell proliferation, angiogenesis, invasion, metastasis and immune elimination and are preponderant on poor prognosis [17, 18].

Although the evidence in support of ongoing dynamic interactions between TAM being instrumental in cancer progression is gradually mounting, the detailed mechanisms and content of communication between macrophages and cancer cells within tumor microenvironment (TME) remain unclear. In this article, we will review the diverse aspects of extracellular vesicles-mediated communications between cancers and TAM, provide a foundation for a potential strategy of cancer treatment by targeting extracellular vesicles or TAM (Table 1).

**Table 1:** Different cancers and TAM communicate though extracellular vesicles

Cancer types & TAM	Exosomal cargo	References
Pancreatic cancer	miR-301a-3p	[15]
	ICAM-1	[16]
TAM	miR-501-3p	[3]
	miR-365	[14]
Gastric cancer	MET	[24]
TAM	ApoE	[4]
	miR-21	[19]
Breast cancer	27-HC	[5]
	miR-375	[29]
TAM	miR-223	[25]
Ovarian cancer	miR-222-3p	[34]
	miR-21-3p	[35]
	miR-125-5p	[35]
	miR-181d-5p	[35]
	miR-940	[36]
Colorectal cancer	miR-25-3p	[7]
	miR-130b-3p	[7]
	miR-425-5p	[7]
	miR-203	[38]
	miR-21	[39]
	Lnc-RPPH1	[40]
TAM	miR-21-5p	[37]
	miR-155-5p	[37]
Glioblastoma	miR-1246	[8]
TAM	miR-21-5p	[42]
Hepatocellular carcinoma	miR-146a-5p	[9]
TAM	miR-125a/b	[43]
Lung cancer	Let-7	[44]

TAM: tumor associated macrophages

## Pancreatic cancer (PCa)

**Fig. 1:**
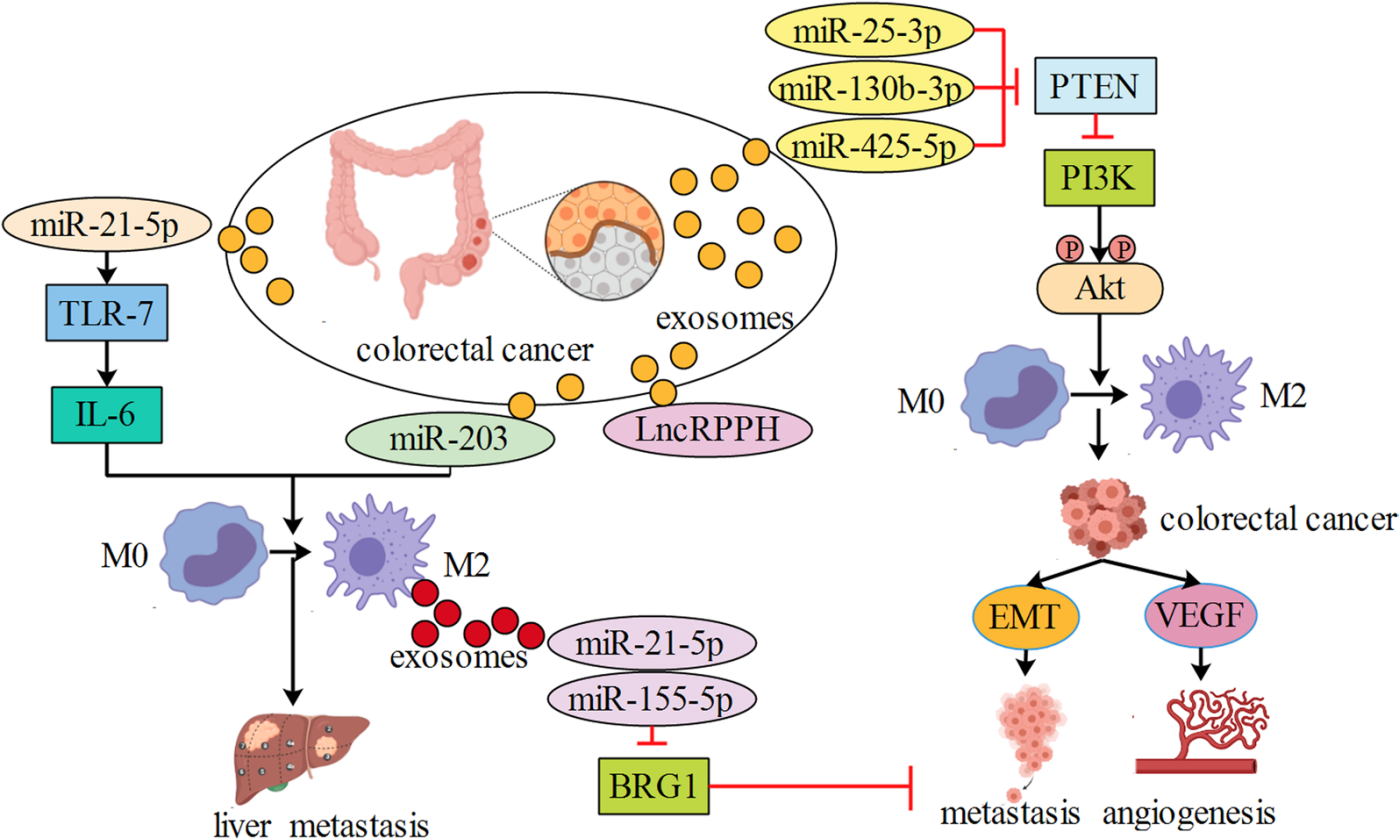
Extracellular vesicles from pancreatic cancer transferred miR-301a and ICAM-1 into macrophages to induce macrophages M2 polarization. M2 macrophages transferred miR-365 and miR-501-3p into pancreatic cancer to promote chemoresistance and metastasis

TGF-β type III receptor (TGFBR3, also known as betaglycan) was found to inactivate TGF-β pathway in cancer formation and metastasis [19, 20]. In PCa, TAM were higher in metastatic tissues than in non-metastatic tissues. TAM-derived exosomal miR-501-3p inhibited tumor suppressor gene TGFBR3 and promoted PCa development by activating the TGF-β signaling pathway [9]. Homeobox D10 (HOXD10) and BH3-like motif-containing protein cell death inducer (BLID) were also potential targets of miR-501, and exosomal transfer of miR-501 conferred doxorubicin resistance and tumorigenesis [21, 22]. Yoav et al. found that transfer of miRNAs in TAM-derived extracellular vesicles could induce gemcitabine resistance in PCa. Specifically, exosomal miR-365 impaired activation of gemcitabine by upregulating the triphospho-nucleotide pool in cancer cells and inducing the enzyme cytidine deaminase; the latter inactivates gemcitabine [23]. Besides, miR-365 showed multiple functions in cancer progression by targeting various genes, and it is therefore desired that further attention be drawn to this field [24–26]. Hypoxic extracellular vesicles derived from PCa cells promoted M2 polarization of macrophages in a HIF1a or HIF2a-dependent manner, which then facilitated the migration, invasion, and epithelial-mesenchymal transition of PCa cells. Exosomal miR-301a-3p derived from PCa cells triggered macrophages M2 polarization by activation of PTEN/PI3K/Akt pathway, which facilitated lung metastasis of PCa in a feed back loop [27]. Runx3, p21, and Smad4 were targeted genes of miR-301a [28, 29], whether these genes are involved in exosomal miR-301a-mediated PCa progression need to be further investigated. Intercellular adhesion molecule-1(ICAM-1), a cell surface glycoprotein, is expressed on various cell types and is involved in cell–cell communication. Extracellular vesicles from ascites-derived human PCa cell line were enriched in ICAM-1, which mediated their successful docking and effective fusing of extracellular vesicles to macrophages. Then, M2-polarized macrophages significantly increased secretion of various tumor-active molecules including MMPs, VEGF, and TNFα to induce tumor progression [30]. While we cannot outline precisely how many proteins may be contributing to exosome absorption and macrophages polarization, it is noteworthy that ICAM-1 would be a promising molecular in future study (Fig. 1).

## Gastric cancer (GC)

TAM were enriched in metastatic patients of GC and could promote migration and invasion of GC cells through secreting extracellular vesicles. Moreover, exosome-mediated intercellular transfer of functional ApoE protein from TAM to GC cells activated PI3K/Akt signaling pathway in recipient GC cells and remodeled the cytoskeleton-supporting migration [10]. In another study, ApoE, a key protein of lipoprotein metabolism, was found to be an anti-angiogenic and metastasis-suppressive factor regulated by multiple miRNAs [31]. This study did not focus on the effects of extracellular vesicles, but it raises the possible that seeking for specific exosomal miRNAs may restore the anti-tumor effects of ApoE. Extracellular vesicles derived from TAM were involved in mediating the resistance of GC to cisplatin, and exosomal miR-21 could be directly transferred from TAM to GC cells, where it inhibited cell apoptosis and activated PI3K/Akt signaling pathway by targeting PTEN [32]. Interestingly, miR-21 was also involved in regulating MAPK/AP-1, CCR7/STAT3/NF-κB pathway [33, 34]. Whether these pathways were significant in exosomal miR-21 mediated cisplatin resistance remains to be investigated. GC-derived extracellular vesicles effectively educated monocytes to differentiate into Programmed cell death 1 (PD1)+ TAM generation. These cells could impair CD8+ T-cell function, thereby creating circumstance that promote GC progression and poor prognosis [35–37]. Since PD-1 plays a significant role in tumor immunity [35–37], immunotherapy combined with targeting PD1+ TAM and tumor-derived extracellular vesicles might restore immune function in GC patients. Helicobacter pylori (H. pylori) is a major risk factor for GC. Remarkably, mesenchymal-epithelial transition factor was increased in extracellular vesicles released from H. pylori-infected GC cells. After absorption, these extracellular vesicles educated macrophages towards a pro-tumorigenesis phenotype and increased IL-1β level via the Akt and MAPK pathways, resulting in migration and invasion in GC [38]. Akt and MAPK pathways are identified as vital tumor regulatory pathways [39–41], investigation into signaling pathways involved in exosome-mediated GC aggression is of impending necessity.

## Breast cancer (BC)

Extracellular vesicles secreted by TAM shuttled invasion-potentiating miR-223 into BC cells, and miR-223 promoted the invasion of BC cells via the Mef2c-β-catenin pathway [42]. Cadherin-6 (CDH6) and long noncoding RNA MEG3 have been respectively recognized as upstream and downstream targets of miR-223 [43, 44], whether these genes were involved in exosomal miR-223-mediated cancer progression remains to be further explored. 27-Hydroxycholesterol (27-HC) is a metabolite of cholesterol. It was found that M2 type macrophages produced higher amounts of 27-HC after being exposed to extracellular vesicles derived from BC. Furthermore, the increased level of 27-HC not only stimulated BC cell proliferation but also promoted the recruitment of CCR2- and CCR5-expressing monocytes by inducing macrophages to express chemokines including CCL2, CCL3 and CCL4 [11]. Another research showed that triple negative breast cancer (TNBC) cells-derived extracellular vesicles stimulated macrophage M2 polarization, creating favorable conditions for lymph node metastasis of TNBC [45]. Extracellular vesicles derived from BC cells transferred miR-375 to TAM through bounding to LDL with CD63 receptor. Additionally, miR-375 directly targeted TNS3 and PXN to increase migration and infiltration of TAM into tumor [46]. This study not only showed the regulatory role of miR-375 but also highlighted its uptake via the CD36 receptor. Indeed, miR-375 is a well-characterized tumor suppressor and have various target genes [47–50]; understanding how BC cells communicate with the macrophages within the tumor microenvironment, especially new uptake-associated markers and target genes, may uncover an avenue for tumor-specific treatment.

## Epithelial ovarian cancer (EOC)

**Fig. 2:**
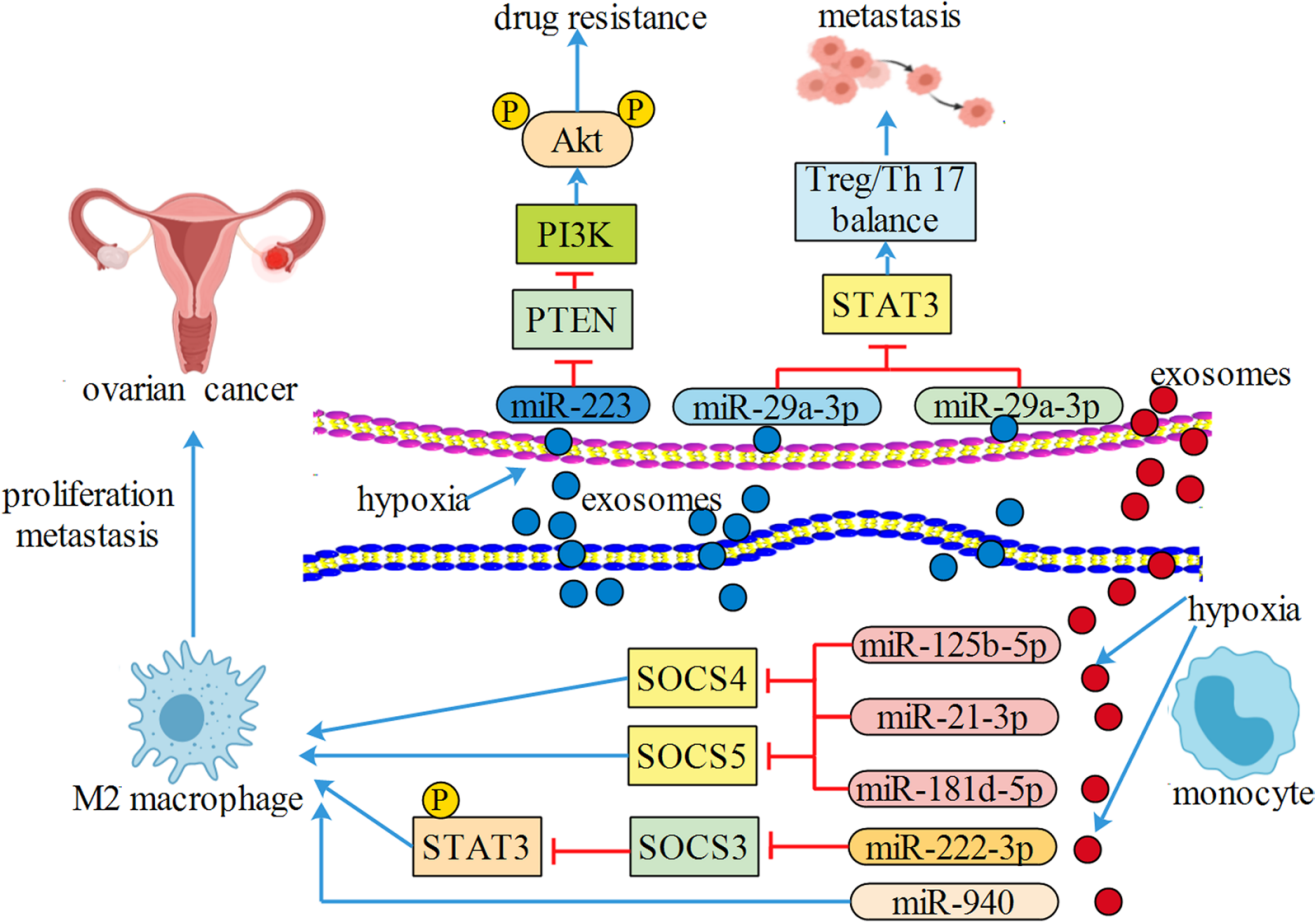
Exosomal miR-125b-5p, miR-21-3p, miR-181d-5p, miR-222-3p and miR-940 from ovrian cancer induced macrophages M2 polarization. Exosomal miR-223, miR-29a-3p and miR-29a-3p from M2 macrophages promoted drug resistance and metastasis

CD4 + T cells have two subsets, regulatory T cells (Treg) and T helper 17 (Th17) cells [51, 52]. The imbalance of Treg/Th17 rate resulted in several tumors including EOC. Zhou et.al found that exosomal miR-29-3p and miR-21-5p from TAM could directly suppress STAT3 and induce Treg/Th17 imbalance, resulting in a higher Treg/Th17 rate and worse patient survival in EOC [53]. TAM-derived extracellular vesicles targeted the miR-146b-5p/TRAF6/NF-κB/MMP2 pathway to suppress human umbilical vein endothelial cell migration; however, EOC-derived extracellular vesicles could transfer lncRNAs to remotely reverse such effect of TAM on endothelial cells [54]. Increasing studies have found that miR-146-5p was involved in tumorgenesis [55–57]. Their studies did not explore the detailed mechanism of lncRNAs, but it raises the possible existence of a signaling axis among exosoma lncRNAs, exosomal miR-146-5p, and target genes. Hypoxic EOC cells triggered macrophages recruitment and induced macrophages M2 polarization; extracellular vesicles derived from hypoxic macrophages increased cell proliferation, decreased apoptosis, and enhanced chemo-resistance of EOC cells. In further research, the authors showed that exosomal miR-223 derived from TAM could be transferred to the co-cultivated EOC cells and promoted the drug resistance of EOC cells via the PTEN-PI3K/Akt signaling pathway [12]. EOC-derived extracellular vesicles activated macrophages to a TAM-like phenotype, and EOC-derived exosomal miR-222-3p triggered macrophages M2 polarization via SOCS3/STAT3 pathway involvement [58]. Their group also found that TAM educated by hypoxic extracellular vesicles derived from EOC cells could promote tumor proliferation and migration in a feedback loop. Through analyzing the extracellular vesicles from hypoxic environment, the authors confirmed higher expressions of miR-21-3p, miR-125b-5p and miR-181d-5p regulating SOCS4/5/STAT3 pathway in TAM [59]. Given that miR-21, miR-125b and miR-181d are oncomiRNAs and strongly related to cancer progression [60–63], more attention could be paid to precisely explore the mechanism. Interestingly, another research confirmed that extracellular vesicles derived from hypoxic EOC could deliver miR-940 to induce macrophages M2 polarization [64]. Indeed, miR-940 has been found to inhibited cancer cell proliferation [65, 66], and how extracellular vesicles selectively pack miR-940 remains to be fully investigated (Fig. 2).

## Colorectal cancer (CRC)

**Fig. 3:**
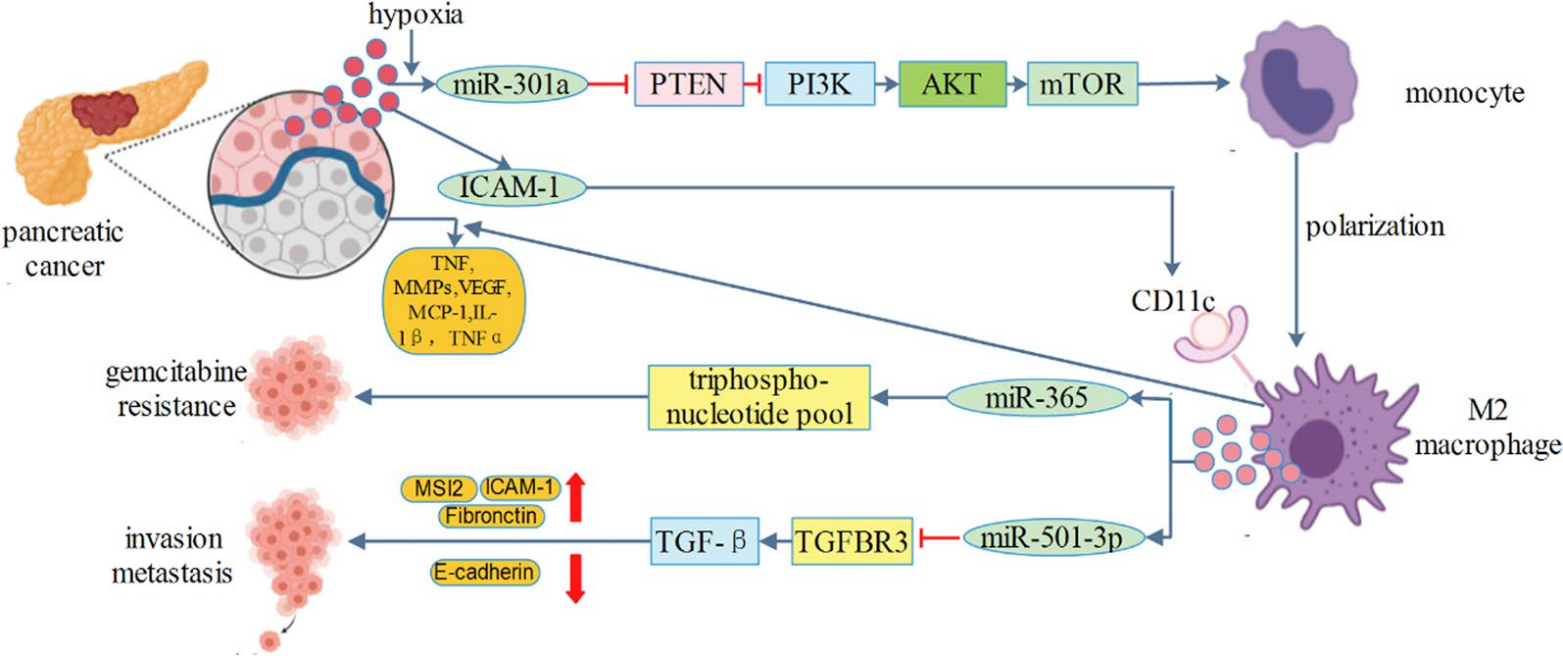
Colorectal cancer secreted exosomal miR-25-3p, miR-130b-3p, miR-425-5p, miR-203 and lnc RPPH to macrophages, resulting in macrophages M2 polarization. M2 macrophages secreted exosomal miR-21-5p and miR-155-5p to colorectal cancer, resulting in metastasis

BRG1 has been identified as a key factor promoting CRC metastasis and participating in cancer progression. Extracellular vesicles from TAM enhanced invasion and migration of CRC cells. Mechanistically, exosomal miR-21-5p and miR-155-5p were transferred from TAM to CRC cells and downregulated expression of BRG1 [67–69]. CRC cells transferred miR-25-3p, miR-130b-3p and miR-425-5p to macrophages via extracellular vesicles. These exosomal miRNAs induced macrophages M2 polarization by regulating PTEN expression through activation of PI3K/Akt signaling pathway. In turn, TAM promoted cancer metastasis by enhancing epithelial-mesenchymal transition and producing vascular endothelial growth factor [13]. Both miR-25-3p and miR-425-5p were two oncogenes [70–73], whereas miR-130b-3p was reported to be a tumor suppressor gene [74, 75]. How these exosomal miRNAs displayed a joint cooperation need to be fully discovered. Another study also found that extracellular vesicles carrying miR-203 from CRC cells were incorporated into monocytes and miR-203 promoted the differentiation of monocytes to TAM [76]. Liver metastasis is commonly observed in CRC patients and is associated with a poor prognosis. Recently, it was found that extracellular vesicles derived from CRC could be specifically targeted to liver tissue and induce liver macrophage polarization toward an interleukin-6 (IL-6)-secreting proinflammatory phenotype through the miR-21/Toll-like receptor 7(TLR7)-IL6 axis [77]. Given that lung and brain are also metastasis sites of CRC, this may raise a hypothesis that CRC-derived extracellular vesicles could induce a distant inflammatory premetastatic niche. Extracellular vesicles derived from CRC cells could transport lncRNA RPPH1 into macrophages which modulate macrophage M2 polarization, thus promoting metastasis and proliferation of CRC cells [78]. Interestingly, CRC cells interacted with macrophages via extracellular vesicles by CAGE-miR-140-5p-Wnt1 axis, thereby regulating autophagic flux and tumorigenic potential [79] (Fig. 3). It is therefore desired that further attention be drawn to this autophagia field.

## Other malignancies

GBM is the most aggressive tumor of brain with a poor median survival, but there are few studies about exosome-mediated interactions between GBM and macrophages. Recently, hypoxic glioma-derived extracellular vesicles were able to induce M2 macrophage polarization, which subsequently promoted glioma proliferation, migration and invasion. Hypoxic glioma-derived extracellular vesicles delivered miR-1246 to induce M2 macrophage polarization by targeting TERF2IP to activate the STAT3 signaling pathway and inhibited the NF-κB signaling pathway [14]. Therefore, miR-1246 may be a novel biomarker for GBM diagnosis and that treatment targeting miR-1246 may contribute to antitumor immunotherapy. In another study, extracellular vesicles with miR-21-5p from TAM increased tumorigenesis and temozolomide resistance in GBM via regulating the expressions of STAT3 and programmed cell death protein 4(PDCD4) [80]. T cell is a key mediator in tumor immunotherapy and the dysfunction of T cell is directly related with various cancers [81–83]. It was found that HCC-derived extracellular vesicles could remodel macrophages by transferring miR-146a-5p, activating NF-κB signaling and inducing pro-inflammatory factors, and resulted in macrophages M2-polarization and T cells inhibition. In particular, the transcription factor Sal-like protein-4 (SALL4) could bind to the promoter of miR-146a-5p, and blocking the SALL4/miR-146a-5p interaction in HCC reversed T cell exhaustion, and delayed HCC progression [15]. Cancer stem cells promote tumorigenesis and progression of HCC, and CD90 is a stem cell marker of HCC stem cells. Recently, extracellular vesicles derived from TAM increased cell proliferation and stem cell properties of HCC cells. Besides, extracellular vesicles derived from TAM expressed lower levels of miR-125a and miR-125b and contributed to cell proliferation and stem cell properties of HCC cell by downregulation of CD90 [84, 85]. Hypoxia is a pivotal environment stressor related to tumor prognosis and progression. Recent study showed that more extracellular vesicles were secreted in lung cancer cells exposed to hypoxia than in those under non-hypoxic conditions. Extracellular vesicles produced by hypoxic lung cancer cells were highly enriched in immunomodulatory proteins and cytokines, which influenced macrophage recruitment and promoted macrophages M2 polarization. Moreover, hypoxia-induced extracellular vesicles enhanced oxidative phosphorylation in macrophages by transferring let-7a, leading to suppression of the mTOR signaling pathway [86].

## Exosome roles in prognosis and treatment

Together, above studies suggest that extracellular vesicles have great potential as delivery vehicles to stimulate and remodel the TAMs in the TME for cancer treatment [87]. It is urgent to identify crucial molecular events linked with the malignant transformation of cancer cells. The present perspective of cancer management is based on the sharply evolving and increasingly comprehensive study on the molecular, genetic, cellular and biochemical basis of cancers. Accumulating evidence implies that extracellular vesicles have versatile functions, indicating their potential applications in future. With its natural ability, the functional extracellular vesicles have vital potential to attenuate cancer metastasis via containing a therapeutic agent and its intracellular cargo. Although regulating exosome cargo, it remains a plausible therapeutic strategy to target its secretion in TME and loading of drugs onto extracellular vesicles. Cancer cell infiltration and their interaction with TME can enhance tumor recurrence and treatment resistance [88]. Extracellular vesicles and sphingolipid carrier characteristics can be used for the diagnosis and prognostic analyses. There are still several unopened questions about their role in TME. Further investigations are warranted for exploring exosomal drugs for cancer treatment. The challenge for the future is to make the best of this knowledge for potential targets for therapeutic intervention and develop more novel biologic therapeutics.

## Conclusions and perspectives

The TME profile of solid malignancies is a tanglesome structure comprising a heterogeneous population of tumor cells, multiple stromal and immune cells, occasionally adipocytes, extracellular vesicles, extracellular matrix proteins, secreted factors and vascular and lymphatic networks [89]. Extracellular vesicles are significant modulators in TME and mediated cellular crosstalk. Extracellular vesicles can influence macrophages polarization through RNAs or proteins, resulting in tumorigenesis and cancer progression. Better understanding of the role of extracellular vesicles and TAM may unveil novel mechanism of cancers to current treatments, further, may provide novel prognostic tools and targets. In summary, the mechanisms of communications between extracellular vesicles and TAM will be useful for development of biomarkers and therapies to improve the treatment outcome. Thus, understanding signaling pathway and machinery among them remains an exciting area for further investigation.

## Data Availability

The data used to support the findings of this study are available from the corresponding author upon request.
